# Pilot Study of Early Adoption of Automated Insulin Delivery in Underresourced Youth

**DOI:** 10.1155/jdr/6886806

**Published:** 2025-11-06

**Authors:** Kevin Yen, Sonali Belapurkar, Cassidy Puckett, Natalie S. Chen, Loren Yglecias, Kavenpreet S. Bal, Jeannie G. Hickey, Lois L. Carelli, Crystal M. Loucel, Maya Lodish, Jenise C. Wong

**Affiliations:** ^1^Division of Endocrinology, Department of Pediatrics, University of California San Francisco, San Francisco, California, USA; ^2^Department of Sociology, Emory University, Atlanta, Georgia, USA

## Abstract

**Background:**

Disparities in outcomes and technology use in children with Type 1 diabetes (T1D) from underresourced backgrounds are well documented. The feasibility of initiating automated insulin delivery (AID) soon after diagnosis of T1D is less certain in this population. This pilot study assessed the feasibility and acceptability of providing access to the Tandem Control-IQ AID system to children with public insurance soon after T1D diagnosis.

**Methods:**

Publicly insured children aged 6–21 years of age within 3 months of T1D diagnosis were eligible for the study. Participants were randomized 2:1 to AID or usual care for 6 months. Continuous glucose monitoring data were collected at baseline, 3 months, and 6 months. Caregivers and youth completed closing surveys and participated in focus group interviews to assess safety and user experience.

**Results:**

Nineteen youth were enrolled, with thirteen in the intervention and six in the control group. The mean age was 11.5 ± 2.3 years, 47% were female, and 89% were from underrepresented racial or ethnic groups. A larger proportion of the AID group compared to the control group achieved the American Diabetes Association benchmark of > 70% time in range (50% vs. 0% of participants at 3 months; 37% vs. 0% of participants at 6 months; not statistically significant). All caregivers and 69% of youth in the AID group reported satisfaction, and 85% of youth continued using AID 6 months after the completion of the study. Focus groups showed favorable experiences with AID use.

**Conclusion:**

Early initiation of AID is feasible and acceptable in youth with recently diagnosed T1D from underresourced populations who historically experience lower technology adoption and less optimal glycemic outcomes. Diabetes clinicians should consider providing tailored support and dedicated resources to families early in diagnosis with T1D to promote AID initiation and continued use.

## 1. Introduction

Diabetes technology has advanced significantly since the first commercially available automated insulin delivery (AID) system was approved in 2016 [1]. The use of diabetes technology, including AID, has become the standard of care for those living with Type 1 diabetes (T1D) [2]. Despite these advances, disparities in the uptake and usage of diabetes devices have been well described [3–7]. Specifically, children with public insurance, from lower income households, and/or who identify as coming from historically marginalized racial or ethnic groups are less likely to use diabetes technology. The drivers behind these inequities are multifactorial and include provider implicit bias, organizational clinical practice, limitations of health insurance coverage, and distrust of technology and healthcare systems [8–10].

While the efficacy of all AID systems has been shown in randomized controlled trials, the feasibility of early adoption of AID is not established in the literature [2]. The American Diabetes Association (ADA) recommends considering starting diabetes technology early in diagnosis, though certain populations of children with T1D may encounter barriers to starting diabetes technology soon after diagnosis. Pivotal trials of AID in children primarily enrolled participants from families with high levels of education, private insurance, and who identify as non-Hispanic White [11, 12]. Prior studies of early initiation of AID have not specifically focused on children from lower income families and/or those who come from minoritized populations [13, 14]. It is important to include diverse populations in AID studies and address inequities given that these families may encounter structural barriers to device use. Strategies to promote early adoption of AID may need to be designed to be culturally sensitive and tailored to unique needs.

The University of California San Francisco (UCSF) Benioff Children's Hospital (BCH) Oakland is a safety net hospital located in the San Francisco Bay Area serving a diverse population of children, about 70% of whom are publicly insured. The diabetes clinic in Oakland provides multidisciplinary care to ~800 children with T1D who identify as Latinx (40%), non-Latinx White (25%), non-Latinx Black (13%), Asian (5%), and other/multiracial (17%). The clinic is staffed with physicians, CDCESs, dieticians, and social workers. At the time of the study (2021–2023), children with T1D in this clinic typically did not start AID until at least 1 year after diagnosis due to limitations of insurance, clinic staffing, or other barriers. Against this background of inequities in diabetes technology use and outcomes, our objectives were to assess the feasibility and acceptability of early adoption of AID among publicly insured children within 90 days of T1D diagnosis in a randomized controlled pilot study, and to illustrate the initiation of AID in marginalized and minority populations, which has not been previously described.

## 2. Materials and Methods

### 2.1. Study Design

This is an open-label randomized controlled pilot study of access to an AID system compared to usual care for newly diagnosed children with T1D with public or no health insurance, for 6 months. All procedures were approved by the institutional review board at the UCSF (IRB Number 20-31736).

### 2.2. Intervention

The integrated t:slim X2 insulin pump with the Dexcom G6 continuous glucose monitoring (CGM) system, running the Control-IQ algorithm (Tandem Diabetes), is an AID system that was FDA-approved for children 6 years of age or older at the time of study [15]. Participants randomized to the intervention were given access to this system within 90 days of T1D diagnosis. They received standard initial and ongoing education from a certified diabetes care and education specialist (CDCES) on the use of the AID system, including the system's benefits and risks, changing pump sets and CGM sensors, and troubleshooting problems.

### 2.3. Usual Care (Control)

Participants in the control arm received standard diabetes education as per usual clinical guidelines. Standard diabetes care included clinic visits at least every 3 months, following guidelines from the ADA. For newly diagnosed patients in our clinic, device technology is introduced on a case-by-case basis by the diabetes care team. The timing of device introduction and initiation is variable and can occur any time after diagnosis. Participants in usual care had the option to start any technology based on the joint decision by the family and their regular diabetes clinician through usual clinical means at any point during the study.

### 2.4. Participants

Potentially eligible participants were identified based on a review of a list of newly diagnosed patients that was maintained by the clinical team. Families were contacted by telephone and/or during the first follow-up visit with the diabetes educator approximately 2 weeks after diagnosis. Eligible children were publicly insured, aged 6–21 years of age, with new-onset T1D within 30–90 days of diagnosis at BCH Oakland between 2021 and 2023. Children who weighed less than 55 lb. and used less than 10 units of insulin per day were excluded from the study based on device manufacturer guidelines. Parental/guardian consent was obtained for all participants under the age of 18 years at the time of study enrollment. All participants under the age of 18 years assented to participate in the study.

### 2.5. Study Procedures

All participants who consented to enroll in the study underwent a 10-day blinded CGM (Dexcom G6 Professional) trial for baseline assessment of glycemic management. Upon completion of the blinded CGM trial, participants were randomized in a 2:1 intervention-to-usual care block whereby the intervention group was started on the AID system. The 2:1 allocation of participants was chosen to enable more participants to potentially benefit from the intervention. Those randomized to usual care were not prohibited from using diabetes technology as part of their standard clinical care. CGM metrics were collected at baseline, 3 months, and end of study (6 months).

AID participants completed study visits at AID initiation, 2, 6, 12, 18, and 26 weeks with the research staff during which information on AID was captured by a brief interview and self-report questionnaire. Participants in both arms were able to contact the research team, including the research CDCESs, at any time by phone or text messaging, for any reason.

Caregivers completed surveys of demographics (race/ethnicity, age, household size, and caregiver level of education) and social vulnerability (food and housing insecurity) at enrollment. Validated instruments measuring psychosocial outcomes were administered at baseline and end of study, which included measures of diabetes-related quality of life (Pediatric Quality of Life Inventory 3.2 Diabetes Module [PedsQL]), Diabetes Impact and Device Satisfaction (DIDS), and diabetes distress (Problem Area in Diabetes-Teen [PAID-T] and Parent Problem Area in Diabetes-Teen [P-PAID-T]) [16–18]. At the study end, caregivers and participants separately completed satisfaction surveys with 5-point Likert scales that assessed their experience with diabetes devices used in the study as well as their experience participating in clinical research.

REDCap (Research Electronic Data Capture) was used for data collection and storage [19]. Glycated hemoglobin (A1c) levels were obtained through a review of the EHR. Device information including time in range (TIR) (70–180 mg/dL), time above range (TAR) (> 180 mg/dL), time below range (TBR) (< 70 mg/dL), and pump delivery/usage were collected from the Tandem research server for the AID group. For the control group, CGM metrics were collected from Dexcom Clarity.

### 2.6. Qualitative Assessment

At study completion, all members of the intervention group (youth and their caregivers) were invited to participate in optional focus groups to discuss their experiences using AID. The purpose of these focus groups was to provide in-depth qualitative data about perceptions of AID use, including benefits and challenges of the technology. All participants in the intervention arm were included, regardless of the amount of time they used AID. Focus groups were held separately for caregivers and for the youth. Focus groups were offered in English or Spanish, but all participants declined the need for a focus group in Spanish or an interpreter. Groups included 2–5 participants per session, lasting 90–120 min, and all sessions were held in person. Groups were facilitated by investigators (K.Y., S.B., and J.C.W.), and the discussion focused on how families learned about device technology, perceptions of using CGM and AID, barriers to and facilitators of use, and desire to continue using the technology in the future.

### 2.7. Statistical Analysis

Stata SE 17 (StataCorp, College Station, TX, United States) was used for statistical analysis. Two sample *t*-tests were used to compare means for normally distributed glycemic and psychosocial outcomes including average blood glucose, TIR, and scores on validated instruments between intervention and control groups. Paired *t*-tests were used to compare changes in these metrics within participants. Chi-square tests were used to compare categorical data. Qualitative data was analyzed using thematic content analysis [20]. Transcripts were reviewed independently by the authors. Constant comparison analysis was used to gather themes across focus groups and amongst the members of the research team [21].

## 3. Results

Thirty-two families were approached for participation in the study. Of those families, 20 participants enrolled in the study, with 14 randomized to the intervention group and six randomized to the control group. One participant in the intervention group dropped out before starting study activities, and a total of 19 participants completed the study. Overall, the mean age of all participants was 11.5 ± 2.3 years. Eighty-nine percent of participants identified with a race or ethnicity other than non-Hispanic White, 37% identified Spanish as the primary language in the home, and 95% of caregivers did not have a college degree. Mean A1c at diagnosis was 13.1% (± 2%). Forty-seven percent of households reported experiencing food insecurity. There were no statistically significant differences in these characteristics between the intervention and control groups (Table 1).

### 3.1. Acceptability

Based on self-report surveys, 69% of individuals in the intervention (AID) group had a positive experience, 23% were neutral, and only one had a negative experience after participating in the study. Six months after study completion, 85% of participants remained on the AID system. Of those who discontinued, the reason given was wanting to take a break from the technology. All 13 caregivers in the AID group reported a positive experience with AID use and would recommend it to families experiencing new-onset T1D. At the end of the study, 12 out of 13 participants in the intervention group chose to continue using AID. Among the usual care group, none of the five participants had started using AID by the end of the study.

### 3.2. CGM Metrics

**Figure 1:**
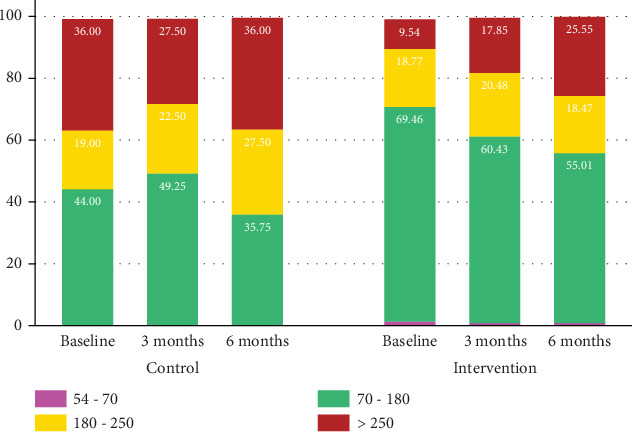
Distribution of continuous glucose monitoring values between the control and intervention groups at baseline, 3, and 6 months.

Comparison of TIR in the control and intervention groups over the 6-month study period showed no significant differences (Figure 1). At baseline, the intervention group had higher TIR (*p* = 0.03). There was a trend toward those in the AID group having higher TIR throughout the study, though overall TIR decreased over the course of the study in all participants.

The number of participants achieving TIR > 70% (ADA benchmark) was not statistically different between groups (Table 2). However, no participant in the usual care group reached the goal of TIR > 70% at any point in the study, compared to 50% and 41% of the AID group at the midpoint and at the close of the study, respectively.

### 3.3. Adverse Outcomes

There were no episodes of diabetic ketoacidosis (DKA) in either the intervention or control group. One participant in the intervention group experienced a pump site infection requiring a visit to the emergency department. Four pumps stopped functioning properly in the intervention group, and these were replaced as soon as possible.

### 3.4. Psychosocial Outcomes

There were no differences in caregiver-reported diabetes-related quality of life, diabetes distress, diabetes impact, and device satisfaction between groups throughout the study (Table 3). Among youth, those in the intervention group reported a statistically significant higher level of diabetes distress at the end of the study compared to those in the control group (Table 4). Both groups reported a trend in reduced diabetes distress over the course of the study. DIDS increased in those in the AID group during the study but decreased in the usual care group (Table 4), although this difference was not statistically significant.

### 3.5. Focus Group Data

Youth and caregivers described benefits and challenges of device use, yet the majority reported a desire to continue using AID despite ongoing challenges. On the positive side, almost all (8 of 10) youth focus group participants noted the benefits of an insulin pump over multiple daily injections, including comfort, reliability, and convenience. At the same time, youth participants (9 of 10) identified an array of challenges in using devices, including difficulties linking their insulin pump and CGM, continued alerts/beeping, disconnection between different devices and their body, concerns about device accuracy, glitches, and issues with charging. When speaking about these concerns, most of these youth participants (9 of 10) specifically linked their concerns to using devices in school, where they may receive less support and/or encounter peer stigma, particularly with device alerts. All nine caregivers agreed with the benefits and challenges that youth reported, particularly in terms of navigating device use at school. However, caregivers also noted that devices made diabetes management more discreet as compared to finger sticks and injections, as long as alarms did not sound. As one caregiver explained, “I think the pump is a really good…[Other] kids look at it, they say like, ‘Oh, it's like a mini phone.' [You] don't get judged because you're pulling out [the pump for] injections.” Another caregiver shared that she felt “the pump, for me, is the best thing that could have happened because now [my son] doesn't really care about the little things like [what people think when he is bolusing insulin before a meal].” At the same time, several caregivers noted that alarms “can be embarrassing” and the continuous signal of difference from peers can make them “feel like they are not normal.”

## 4. Discussion

The ADA and the International Society of Pediatric and Adolescent Diabetes highlighted the benefits and importance of AID in their latest guidelines while recognizing the disparities that exist in the use of advanced AID [2, 22]. Despite this, few studies have examined the experience of children with T1D who are from historically underresourced communities in initiating AID soon after diagnosis. To our knowledge, this is the first study to assess the feasibility of using AID in new-onset T1D among those who are publicly insured. Our results showed overwhelming acceptability of early adoption of AID. Additionally, no participants had any major adverse events, including DKA or severe hypoglycemia, that required an emergency room visit or hospital stay. The overall safety of our study is significant since a common reason healthcare practitioners may hesitate to recommend technology early in diagnosis is concern with the ability of new-onset families to use devices safely [8]. Healthcare teams may worry that families are at risk for severe complications because of improper device use, but our results suggest that these devices may be used safely with proper guidance. The acceptability and safety of use are also highlighted in qualitative data where both youth participants and their families reported very positive attitudes toward and trust in AID.

Previous studies have shown across different AID systems that the use of AID improves TIR in both children and adults [23–26]. While glycemic metrics were not statistically different between the control and intervention groups in our feasibility study, it is noteworthy that 40% of participants in the AID group achieved the difficult-to-attain ADA goal of TIR > 70%, while no participants in the control group achieved this goal. It is likely that the small sample size in this feasibility and acceptability pilot study did not have enough statistical power to show differences in glycemic metrics, which were considered to be secondary outcomes. The small sample size likely contributed to the statistically significant difference in TIR at baseline between the AID and control groups. This makes it difficult to draw meaningful conclusions regarding the impact of early AID initiation on TIR in early T1D. Larger scale studies are needed to address this very important question. Interestingly, recent studies reporting real-world data on glycemic metrics in the general population state that about 36% of youth using AID reach glycemic targets, which is remarkably similar to our cohort [27].

Another potential clinical concern about introducing AID technology early in diagnosis is that AID may have a negative effect on psychosocial measures. In our study, there was no statistically significant difference in caregiver diabetes distress and diabetes-related quality of life between AID users and those receiving usual care. This supports the hypothesis that the introduction of diabetes technology early in diagnosis is feasible, acceptable, and does not lead to overwhelming distress in our cohort. Qualitative data from caregivers suggest that while there is a learning curve when starting and using diabetes technology, the benefits largely outweigh the challenges.

For children with T1D, using technology is not always positive and may contribute to diabetes distress. Children in the AID group had higher levels of diabetes distress compared to the control group. Consistent with this, qualitative data showed frustration with alarms was a recurring theme. These safety features often disrupt the classroom and lead to embarrassing moments for children amongst their peers. It is possible that these factors might have contributed to some participants in the AID group wanting to pause use of the system at the end of the study. Healthcare teams and device manufacturers should continue to consider the patient perspective on using technology in real-world settings, developing age-appropriate, user-informed education and design in newer iterations of device technology.

We recognize that there are several components of our study that differ from real-world use of diabetes technology. As this is a research study with voluntary participation, there is self-selection among our participants, where willingness to participate in the trial may suggest inherent propensity and openness to diabetes technology. It is possible that those who chose not to participate in this study might have had less positive experiences with AID use early in diagnosis. There were a few eligible, potential participants who were ultimately excluded from the study because they were unable to complete enrollment activities prior to the 90-day post new-onset diabetes diagnosis, as specified by the study protocol. The reasons for delay in enrollment included inability to complete the blinded CGM trial and/or complete consent and baseline surveys remotely within 90 days of diagnosis. If structural and systematic barriers exist that preclude inclusion in a clinical study early in diagnosis, perhaps these same factors may contribute to the coordination necessary to start device technology in this recent onset time period. Finally, outside of a research study setting, additional barriers exist to timely device initiation. These include the time needed to make an informed decision about which type of pump or AID system to use, the requirement for some insurance companies to attend a comprehensive pump class or education session prior to approving the prescription, limited availability of classes and educators for immediate education and training, and other social drivers that may delay clinic attendance and efficient distribution of pump supplies. As diabetes teams consider starting AID systems early in diagnosis, clinics will need to explore ways to overcome these barriers in an equitable manner.

Lastly, we provided more frequent touchpoints with study participants as compared to usual care. In addition to study visits, study CDCESs had direct communication with families via phone and text messaging outside of business hours, often to help troubleshoot the devices. The positive experience in our study may be related to the close relationship with the educator team in addition to the use of the devices. This raises the possibility that success with early initiation of technology lies not only in the availability of the devices but also in the nature of the support system. Our study did offer means of communication and support outside of the traditional paradigm of diabetes care largely due to restrictions on in-person and face-to-face meetings during the COVID-19 pandemic. For example, we utilized texting as a means of communication and sending screenshots of CGM data or pump screens for troubleshooting. Meetings conducted on secure, video-based platforms were used frequently for study check-in. We also used the pump manufacturer's mobile app, which automatically uploaded data to the cloud-based software, for sharing and reviewing diabetes data to minimize in-person downloads. Utilization of these less conventional methods of care delivery may be essential for early adoption of diabetes technology at full scale. This is especially true for underresourced populations who may not have the ability to contact clinics during regular business hours due to demanding work schedules and/or family responsibilities.

## 5. Conclusion

This pilot demonstrated the safety, feasibility, and acceptability of early adoption of diabetes technology in underresourced youth. As diabetes technology evolves and devices become more complex, our experience through this study highlights the need to safely and efficiently deliver advanced diabetes technology at scale in diverse, real-world populations.

## Figures and Tables

**Table 1:** Participant characteristics.

	**Intervention**	**Control**
*n*	13	6
Female	6 (46%)	3 (50%)
Age (years)	11.5	11.5
Race/ethnicity		
Hispanic other	5 (38%)	4 (66%)
Hispanic White	1 (7.5%)	1 (17%)
Non-Hispanic Black	3 (23%)	1 (17%)
Non-Hispanic White	2 (15%)	0
Asian	1 (7.5%)	0
Hispanic Black	1 (7.5%)	0
A1c at diagnosis (%)	13.3	12.8
Caregiver with college degree	1 (7.5%)	0
High school or above (caregiver)	8 (62%)	2 (33%)
Food insecurity	7 (54%)	2 (33%)
English not primary at home	3 (23%)	4 (66%)

Abbreviation: A1c, glycated hemoglobin.

**Table 2:** Achievement of continuous glucose monitoring goals (TIR > 70%).

	**Intervention**	**Control**	**p**
*n* (%)			
Baseline	8 (61.5%)	2 (33%)	0.49
3 months	5 (50%)	0	0.074
6 months	5 (41.6%)	0	0.119

Abbreviation: TIR, time in range.

**Table 3:** Change in psychosocial outcomes in youth.

**Survey**	**Interpretation**	**Intervention**		**Control**	
		**Baseline**	**Closing**	**Baseline**	**Closing**
PedsQL	Higher is better quality of life	63.4	65.7	60.7	76.7
PAID-T	Higher is more distress	64.8	61.4^a^	53.5	34.5
DIDS: device satisfaction	Higher is more satisfaction	3.6	4.7	3.8	3.6
DIDS: diabetes impact	Higher is more anxiety/worry/impact	3.9	4.1	4.4	3.3

Abbreviations: DIDS, Diabetes Impact and Device Satisfaction; PAID-T, Problem Areas in Diabetes-Teen; PedsQL, Pediatric Quality of Life Inventory 3.2 Diabetes Module.

^a^Difference was statistically significant (*p* = 0.02) at closing between the control group and intervention group.

**Table 4:** Change in psychosocial outcomes in caregivers.

**Survey**	**Interpretation**	**Intervention**		**Control**	
		**Baseline**	**Closing**	**Baseline**	**Closing**
PedsQL	Higher is better quality of life	67.2	66.7	67.6	71.9
P-PAID-T	Higher is more distress	42	42	33.2	33.2
DIDS: device satisfaction	Higher is more satisfaction	7.2	7.4	7.5	8.1
DIDS: diabetes impact	Higher is more anxiety/worry/impact	3.5	3.7	3.5	3.9

Abbreviations: DIDS, Diabetes Impact and Device Satisfaction; P-PAID-T, Parent-Problem Areas in Diabetes-Teen; PedsQL, Pediatric Quality of Life Inventory 3.2 Diabetes Module.

## Data Availability

The data are available on request from the authors.
